# Modification of Surface Hydrophobicity of PLA/PE and ABS/PE Polymer Blends by ICP Etching and CF_x_ Coating

**DOI:** 10.3390/ma13235578

**Published:** 2020-12-07

**Authors:** Vedrana Lovinčić Milovanović, Cédric Guyon, Ivana Grčić, Michael Tatoulian, Domagoj Vrsaljko

**Affiliations:** 1 MAXICON Inc., Kružna 22, 10000 Zagreb, Croatia; 2 Chimie ParisTech, PSL University, CNRS, Institut de Recherche de Chimie Paris, 75005 Paris, France; cedric.guyon@chimieparistech.psl.eu (C.G.); michael.tatoulian@chimie-paristech.fr (M.T.); 3 Faculty of Geotechnical Engineering, University of Zagreb, 42000 Varaždin, Croatia; ivana.grcic@gfv.hr; 4 Faculty of Chemical Engineering and Technology, University of Zagreb, 10000 Zagreb, Croatia

**Keywords:** acrylonitrile/butadiene/styrene, microreactors, polylactide, 3D printing, surface hydrophobicity

## Abstract

The flow regime inside the channel of 3D printed microreactors is defined by the surface properties of the channel walls. Polylactide (PLA) and acrylonitrile/butadiene/styrene (ABS) are two polymers that are the most common in additive manufacturing using fused filament fabrication, commonly known as “3D printing”. With the aim of developing new materials for the 3D printing of microreactors whose channel surface hydrophobicity could be modified, PLA and ABS were blended with cheaper and widely used polymers-high-density polyethylene (PE-HD) and low-density polyethylene (PE-LD). Polymer blend surfaces were treated with inductively coupled plasma (ICP) and coated by fluorocarbon-based material (CF_x_) plasma deposition treatment in order to modify surface hydrophobicity. It has been shown that the modification of surface morphology of PLA polymer blends can be achieved by ICP etching and CF_x_ coating, while this was not possible for ABS polymer blends under the conducted treatment conditions. The treated surface of PLA/PE-HD 90/10 showed a contact angle of 121.6° which is 36° higher than the contact angle measured on the untreated surface. Surfaces that have achieved contact angles higher than 120° have an “island like” surface morphology. Samples with higher “islands” showed higher contact angles, that confirmed that the hydrophobicity also depends on the height of the “islands”. Furthermore, it has been found that etching time significantly impacts the contact angle values and surface morphology of the PLA polymer blends, while the CF_x_ coating time does not have significant impact on the surface properties.

## 1. Introduction

Nowadays, superhydrophobicity of surfaces is a desirable property in many products and technologically important materials. Reduction in water resistance on ships and surface protection of equipment in contact with water are unthinkable without hydrophobic coatings. Hydrophobic impregnation is the basic protection method of concrete bridges, silos, buildings and walls and it is applied in order to prevent the penetration of water into the concrete structure. Prescription eyeglasses with a hydrophobic layer to prevent the retention of raindrops, waterproof textiles, facade paints and nano-polymer coatings on cars for reduced adhesion and self-cleaning during rain, are examples of today’s wide-ranging applications of surface superhydrophobicity properties in everyday life. The inspiration for the development of such products comes from nature. One of the most famous examples of a plant with superhydrophobic surface properties is the lotus flower (lat. *Nelumbo nucifera*).

**Figure 1:**
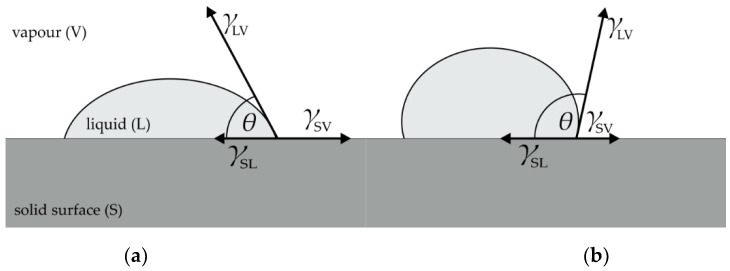
Contact angles of liquids on (**a**) non-hydrophobic and (**b**) hydrophobic surface.

The term superhydrophobicity is used for hydrophobic surfaces where the contact angle with water is greater than 150°. The contact angle (usually identified as *θ*) is an angle between the solid surface and the tangent placed on the droplet from the point where the three intermediate surfaces meet (solid–liquid, liquid–vapor and solid–vapor), as shown in Figure 1.

A flat solid surface contact angle can be derived from Young’s Equation (1) as cos*θ* (2):

(1) $$\gamma_{\mathrm{SL}}\;+\;\gamma_{\mathrm{LV}}\;\cos\theta \;=\;\gamma_{\mathrm{SV}}$$

(2) $$\cos\theta \;=\;\frac{\gamma_{\mathrm{SV}}\;-\;\gamma_{\mathrm{SL}}}{\gamma_{\mathrm{LV}}}$$

where *γ*_SL_ is surface tension (free surface energy) that occurs at the solid–liquid interface, *γ*_LV_ is the surface tension (free surface energy) at the liquid–vapor interface and *γ*_SV_ is the surface tension (free surface energy) at the solid–vapor interface. If the contact angle is less than 90°, the liquid will spill and cover the solid surface. It is considered as a good wetting property and also a hydrophilic property of the surface when the liquid is water. If the contact angle is greater than 90°, the liquid remains on the limited surface in the form of a droplet. It is considered as poorly wetting or not wetting property, as well as a hydrophobic property of the surface when the liquid is water.

**Figure 2:**
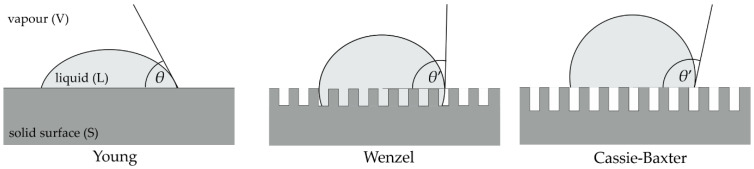
Behavior of liquid on smooth and rough surface.

However, Young’s equation is applicable only to a smooth and flat surfaces. When a droplet comes in contact with a rough surface, the droplet can completely cover the surface, thus increasing the interface that is in contact with the liquid, as described by Wenzel [1]. It can also cross over rough parts of the surface and leave the interfaces of the solid–liquid and liquid–vapour beneath, as suggested by Cassie and Baxter [2,3]. Figure 2 shows the behavior of a liquid on a smooth surface and the proposed models of behavior on a rough surface.

When the liquid completely covers the rough surface, Wenzel [1] proposed a modification of Young’s equation to calculate the contact angle *θ*’ taking into account the surface roughness factor, *r* (3).

(3) $$\cos\theta^{\prime }\;=\;\frac{r\;\left(\gamma_{\mathrm{SV}}\;-\;\gamma_{\mathrm{SL}}\right)}{\gamma_{\mathrm{LV}}}\;=\;r\cos\theta$$

The surface roughness factor represents the ratio of the real area of the rough surface and the geometric projection of the same surface. *θ* represents the contact angle at the smooth and flat surface. According to Wenzel’s rough surface model, roughness enhances the surface property towards stronger hydrophilicity of the hydrophilic surface or towards stronger hydrophobicity of hydrophobic surface [4].

For the other model, when the droplet crosses over rough parts of the surface and leaves the trapped vapour beneath, Cassie and Baxter [2,3] suggested a modification of Young’s equation for calculating the contact angle *θ*’, taking into account the proportion of the solid surface in contact with the liquid, *f*_S_ (4):

(4) $$\cos\theta^{\prime }\;=\;\frac{f_{S}\;\left(\gamma_{\mathrm{SV}}\;-\;\gamma_{\mathrm{SL}}\right)}{\gamma_{\mathrm{LV}}}\;-\;(1\;-\;f_{S})\;=\;f_{S}\cos\theta \;-\;(1\;-\;f_{S})$$

where (1 − *f*_S_) represents the portion of liquid in contact with vapour and *θ* represents the contact angle at the smooth and flat surface. Although the roughness factor *r* is not directly present in the Cassie–Baxter Equation (4), the Cassie–Baxter model assumes that the surface is topographically complex and the surface is considered rough.

Superhydrophobic surfaces (*θ* greater than 150°) are extremely difficult to wet and they show a self-cleaning effect [5]. It has been shown that the self-cleaning effect develops on surfaces that have low surface free energy, high water contact angle and high surface roughness, i.e., complex surface morphology [5].

When choosing materials for production of microreactors by 3D printing, it is important to take into account that the flow in microchannels depends on the interactions of molecules with the solid surface. Therefore, surface hydrophobicity, and particularly superhydrophobicity, is a desirable surface property of the microreactor.

Polylactide (PLA) and acrylonitrile/butadiene/styrene (ABS)—the most commonly used polymers for 3D printing—do not show surface superhydrophobicity. Modification of the surface properties of polymers can be achieved by blending two different polymers and by treatment of the polymer blends [6,7,8,9,10,11,12,13,14]. To achieve superhydrophobicity of the surface on different metal and polymer surfaces, various treatments can be applied, such as sol-gel reactions [15,16,17], electrochemical deposition [18,19,20], layer-by-layer deposition [21,22] and spin-coating [23]. Each of these methods has its advantages and disadvantages and one of the most promising techniques for the development of superhydrophobic surfaces is plasma etching [24,25,26]. Plasma etching is a dry and precise technique. It is a selective technique where usually one phase or compound reacts more quickly with the plasma, so the etching rate is different at different places on the surface [25]. 

Improvement of the hydrophobicity of PLA polymer blends has already been investigated and it has been shown that a change in the surface of the polymer blend and specific surface roughness can be achieved by plasma etching techniques and surface post-treatment [27,28]. According to the available literature, no research of this type has been performed with ABS polymer blends. The effects of etching on the ABS surface were investigated back in 1968 [29], but the etching was carried out as a wet process with the mixture of chromium-sulfuric acid. Research has shown that the chemical acts selectively on rubber particles (polybutadiene), while the matrix remains largely unchanged. Additionally, it has been shown that larger rubber particles are more susceptible to etching, while small particles remain almost unchanged [29]. The research on the possibilities of etching the polymer blends with ABS was carried out on polymer blend of thermoplastic polyurethane elastomer (TPU) and ABS [30]. The etching was also carried out as a wet process where methyl ethyl ketone (MEK) was used for surface treatment. Research has shown that the duration of etching is very important. SEM micrographs of the sample have shown that after 2 h of etching, the morphology of the surface is very similar to the untreated sample, after 3 h of etching cavities are clearly visible indicating the morphology of the dispersed ABS phase in the TPU matrix, while after 4 h the sample is unsuitable for SEM so determination of morphology could not be obtained.

The flow regime inside the channel of every microreactor is defined by the surface properties of the channel walls. With the aim of developing new materials for 3D printing of microreactors, whose channel surface hydrophobicity could be modified, PLA and ABS based materials are studied. More specifically, this research investigated possibility of surface hydrophobicity modification of PLA/low-density polyethylene (PE-LD), PLA/high-density polyethylene (PE-HD), ABS/PE-LD and ABS/PE-HD polymer blends prepared in 95/10, 90/10 and 80/20 weight ratios. Polymer blend surfaces were treated with inductively coupled plasma (ICP) and coated with fluorocarbon-based material (CF_x_) plasma deposition treatment. Polymer weight ratios were selected based on previous research where significant modification of hydrophobicity was achieved on PLA/PE-LD polymer blend exactly in 90/10 ratio [28]. “Island like” morphology was created on the surface and increased hydrophobicity was obtained.

## 2. Materials and Methods

Polymers used for the investigation were: polylactide (PLA), Ingeo™ 4043D (NatureWorks LLC, Blair, NE, USA), designed for injection molding applications; acrylonitrile/butadiene/styrene (ABS), Magnum^TM^ 3504 (Trinseo Europe GmbH, Samstagern, Switzerland); low-density polyethylene (PE-LD), DOW™ LDPE 780E (The Dow Chemical Company, Horgen, Switzerland) and high-density polyethylene (PE-HD), 2004 TN 52 (Total Petrochemicals, Saint-Avold, France).

Polymer blends were mechanically mixed using twin-screw extruder (Rondol 21 mm LAB TWIN) (Rondol Technology Ltd., Stoke on Trent, United Kingdom) at a rotational frequency of 50 rpm. Polymer blends with PLA were prepared at a temperature of 160 °C while the ABS polymer blends were prepared at a temperature of 180 °C due to the higher melting temperature of ABS compared to PLA. Polymer components were mixed in a vessel and added in the extruder. Extruded blends were cut up in small pieces and 1 mm thick sheets were formed by hot pressing. Hot pressing was performed with 11 MPa pressure, at 190 °C with preheating time 3–4 min and heating for 5 min on a Dake hydraulic press (Dake, Grand Haven, MI, USA). Fontijne press (Fontijne Presses, Delft, The Netherlands) was used to cool the sheets to the room temperature. Polymer blends were prepared in weight ratios 95/5, 90/10 and 80/20.

In order to increase the hydrophobicity by changing the surface topography of two-component polymer blends, polymer blends and pure polymers were etched using inductively coupled argon plasma (ICP) and treated with a fluorocarbon-based Teflon-like coating (CF_x_).

ICP etching was conducted in a Hybrid Plasmionique reactor (Plasmionique Inc., Varennes, QC, Canada) under the process conditions: 2.5 kV, 75 W, different time of processing as shown in Table 1, *p* = 5.4 × 10^−4^ Torr = 7.2 × 10^−2^ Pa, *p* (before treatment) = 7 × 10^−6^ Torr = 9.3 × 10^−4^ Pa.

CF_x_ coating was performed in a FLR300-H Plasmionique PECVD reactor (Plasmionique Inc., Varennes, QC, Canada) under the process conditions: *V* (CF_4_) = 84 cm^3^ min^−1^, *V* (H_2_) = 5 cm^3^ min^−1^, 35 W, different time of processing as shown in Table 1, *p* = 260 mTorr = 35 Pa, *p* (before treatment) = 1.7 × 10^−6^ Torr = 2.3 × 10^−4^ Pa.

Contact angle of the droplet at the three-phase boundary was measured by sessile drop method at 23 °C using an OCA 20 goniometer (DataPhysics Instruments, Filderstadt, Germany). Deionized water (*κ* = 2.0 μS cm^−1^) with a droplet volume of 3 µL was used. Five contact angle measurements were performed on each sample.

Morphology was studied by the scanning electron microscope (SEM) on the surfaces of selected samples. VEGA 3 Tescan scanning electron microscope (Tescan, Brno, Czechia) was used. All the samples were gold sputtered prior to SEM characterization.

Heights of the islands (relative to the surrounding surface) were determined by vertical scanning interferometry (VSI). Wyko NT9100 device (Veeco, Plainview, NY, USA) was used for the research. The height of the island was determined by measuring the height difference between the middle and at the edge of the island. The middle and edge of the “island” were determined visually.

## 3. Results and Discussion

Previous research on PLA/PE-LD and PLA/PE-HD polymer blends, where thermal properties of the selected samples were determined by the differential scanning calorimetry (DSC) and SEM characterization were conducted, has shown that PLA and PE-LD as well as PLA and PE-HD in 90/10 ratio are both immiscible blends, with high crystallization degree and no interaction between dispersed (polyethylene) phase and the matrix (PLA) [31].

**Figure 3:**
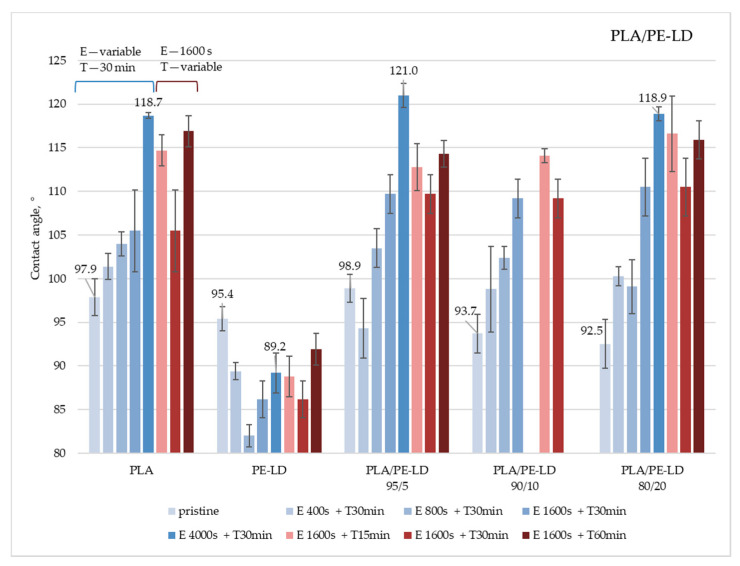
Water contact angles on Polylactide (PLA)/low-density polyethylene (PE-LD) polymer blends treated under different etching and coating conditions (E—etching; T—CF_x_ coating).

**Figure 4:**
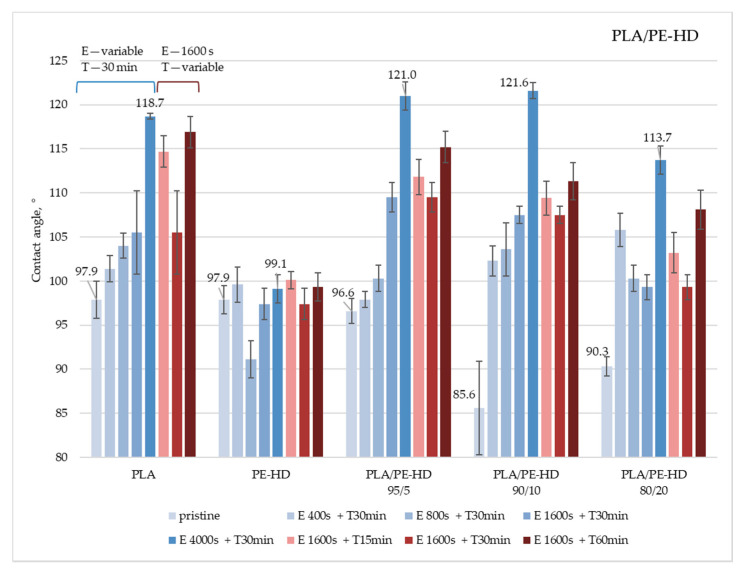
Water contact angles on PLA/high-density polyethylene (PE-HD) polymer blends treated under different etching and coating conditions (E—etching; T—CF_x_ coating).

The contact angle, as a surface property that indicates the surface hydrophobicity, was determined by the sessile drop method. The measurements were performed on untreated samples and samples treated by etching and coating with CF_x_. Obtained results for the polymer blends PLA/PE-LD and PLA/PE-HD are given in Figure 3 and Figure 4. All data can be found in Supplementary Materials—Table S1.

The obtained results show that hydrophobicity of pure PLA can be increased with ICP etching and CF_x_ coating. Unlike the PLA, results for the pure PE-LD sample show that higher contact angle is not achieved after any of the applied treatments. Such results suggest that PE-LD should be less etched than PLA in PLA/PE-LD blends so the “island like” structure could be expected. Results for PLA/PE-LD polymer blends (Figure 3) show that almost all treated samples of PLA/PE-LD polymer blends have a higher water contact angle than the untreated samples, as expected. Additionally, it is important to notice that an increase in processing time, regardless of the applied treatment—etching or CF_x_ coating—does not necessarily lead to an increase in the water contact angle. The best results on all PLA/PE-LD samples are achieved by etching for 4000 s and CF_x_ coating for 30 min. The largest modification in hydrophobicity, when comparing contact angles of untreated and treated sample, is achieved on a PLA/PE-LD 95/5 sample treated by etching for 4000 s and CF_x_ coating for 30 min, achieving a contact angle difference of 22.1°. The highest contact angle, 121.0°, is measured on the same sample (PLA/PE-LD 95/5). Even though the trend for all treated PLA/PE-LD samples shows an increase in water contact angle, the PLA/PE-LD 95/5 sample failed to achieve a higher contact angle after etching for 400 s and CFx coating for 30 min. Such behavior can be considered as an experimental error.

Similar results were obtained for PE-HD and PLA/PE-HD samples (Figure 4). The highest increase in the contact angle, i.e., increase in the hydrophobicity, in all samples is achieved by etching for 4000 s and CF_x_ coating for 30 min. The largest modification in hydrophobicity, comparing the contact angle for untreated and treated samples, is achieved on a PLA/PE-HD 90/10 sample treated by etching for 4000 s and CF_x_ coating for 30 min, achieving a contact angle difference of 36.0°. The highest contact angle, 121.6°, is measured on the same sample (PLA/PE-HD 90/10). Furthermore, it can be concluded that surfaces coated for 60 min show the highest contact angle, compared to the surfaces coated for shorter times.

The obtained results for both PLA/PE-LD and PA/PE-HD polymer blends indicate that surface morphology is modified in a way that PLA is more etched than the PE so the surface is topographically complex and the surface can be considered rough.

**Figure 5:**
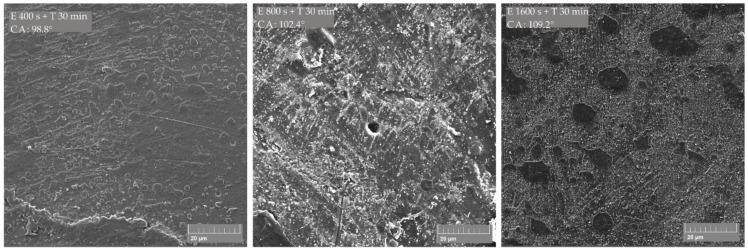
SEM micrographs of PLA/PE-LD 90/10 polymer blend treated by etching (E) and CF_x_ coating (T); measured contact angle is indicated as CA.

**Figure 6:**
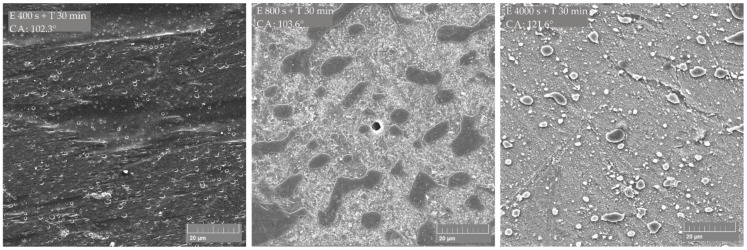
SEM micrographs of PLA/PE-HD 90/10 polymer blend treated by etching (E) and CF_x_ coating (T); measured contact angle is indicated as CA.

These findings are confirmed with the SEM micrographs given in Figure 5 and Figure 6. In our previous research [31], SEM micrographs have shown that domains of PE-LD, as well as PE-HD were dispersed in the PLA matrix and PLA was dispersed in PE matrix when PE amount was higher. These results indicated an immiscibility of the two phases. Blends with dispersed PLA domains in PE matrix have shown the “crater like” structure after the etching, and the CF_x_ coating did not change the water contact angle significantly. On the contrary, blends with dispersed PE domains in PLA matrix had shown the “island like” structure after the etching, and the CF_x_ coating changed the water contact angle significantly. For this reason, in this research “island like” structures were expected as a result of different etching rates on the different phases. 

SEM micrographs of the PLA/PE-LD 90/10 polymer blend (Figure 5) show the formation of an “island like” structure where the matrix (PLA) is more etched and the dispersed domain (PE-LD) is less etched and stands like an island above the matrix level. Such an “island like” structure is produced already with etching for 400 sec and CF_x_ coating for 30 min, but it seems that the island does not reach a sufficient height to significantly increase the contact angle. The measured contact angle on the treated surface was 98.8°, which is 5.1° higher than on the untreated surface. When etched for 1600 s and CF_x_ coated for 30 min, the islands visually appear higher than the surrounding matrix and a higher contact angle (109.2°) is achieved. Island size ranges from 2 μm to 20 μm, and 6 μm sized islands are the most common.

SEM micrographs of the PLA/PE-HD 90/10 polymer blend (Figure 6) show similar surface morphology like the PLA/PE-LD 90/10 blend. The “island like” structure is gained after etching for 800 s and CF_x_ coating for 30 min and the measured contact angle is 103.6°. The highest contact angle achieved in this study is 121.6°. It is achieved on a surface with the 4 μm to 8 μm wide islands, and the 6 μm wide islands are the most common. The distance between islands ranges from 10 μm to 20 μm. This surface structure is achieved by etching for 4000 s and CF_x_ coating for 30 min.

**Figure 7:**
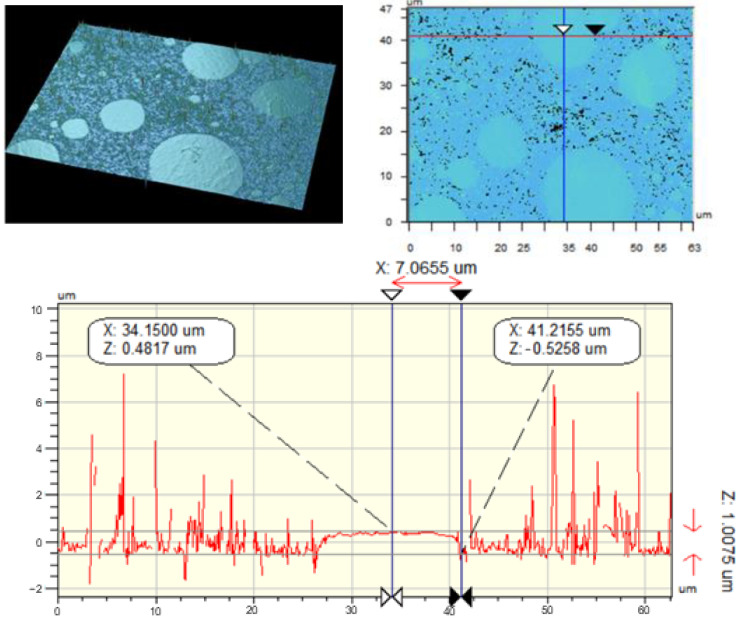
Results of the vertical scanning interferometry (VSI)-pictures of the surface and height profile of the sample PLA/PE-HD 80/20 E 4000 s + T 30 min.

In order to compare the island heights determined by SEM, a series of untreated and treated surfaces of the PLA/PE-HD 80/20 polymer blend were scanned by vertical scanning interferometry (VSI). This technique is based on white light interferometry, where shallow depths of surface are in focus so the surface must be scanned/focused over the whole vertical height to get 3D model. Interferograms consist of fringes (light and dark bands) that represent the points of the same height. Achieved interferograms are digitally transformed into the topographic 3D model from which the surface height can be determined [32]. In this research the height of the island is determined by measuring the height difference between the middle and at the edge of the island. The middle and edge of the island are determined visually. Results show that the height of islands ranges from 0.1978 µm to 1.0075 µm, as shown in Figure 7.

**Figure 8:**
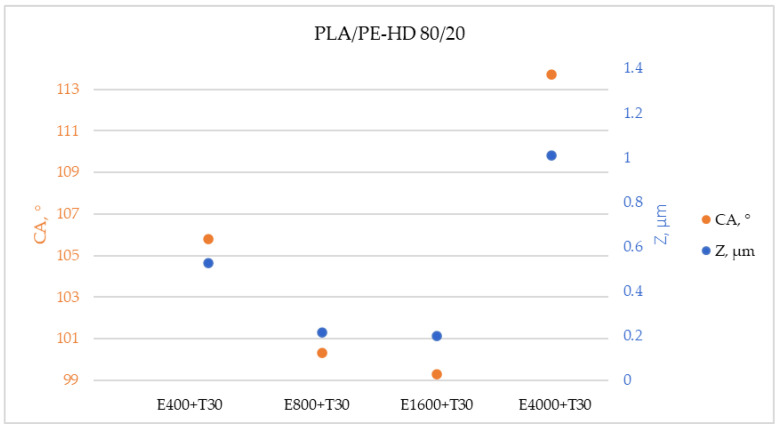
Results of the vertical scanning interferometry (VSI)-trend of contact angle and island height change in relation to applied treatment on PLA/PE-HD 80/20 (E—etching in s, T—CF_x_ coating in min, CA—contact angle, Z—island height).

Obtained results for PLA polymer blends do not deviate from previous studies where it has been revealed that the contact angles, that can be related to hydrophobicity, were achieved at the “island like” structures with the island height of 0.2 µm [28], i.e., at the surface with the pillar structures up to 30 µm high [33]. Furthermore, if the measured contact angles and measured heights of the island are shown on the same graph, the same trend is determined (Figure 8). Additionally, higher water contact angles were measured on the surfaces with higher islands. This confirms the conclusions of previous research that the hydrophobicity of the surface also depends on the height of the islands.

**Figure 9:**
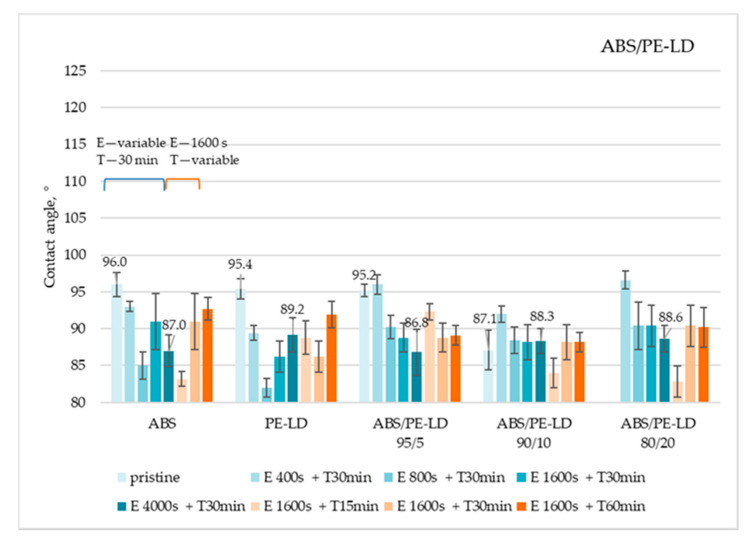
Water contact angles on ABS/PE-LD polymer blends treated under different etching and coating conditions (E—etching; T—CF_x_ coating).

**Figure 10:**
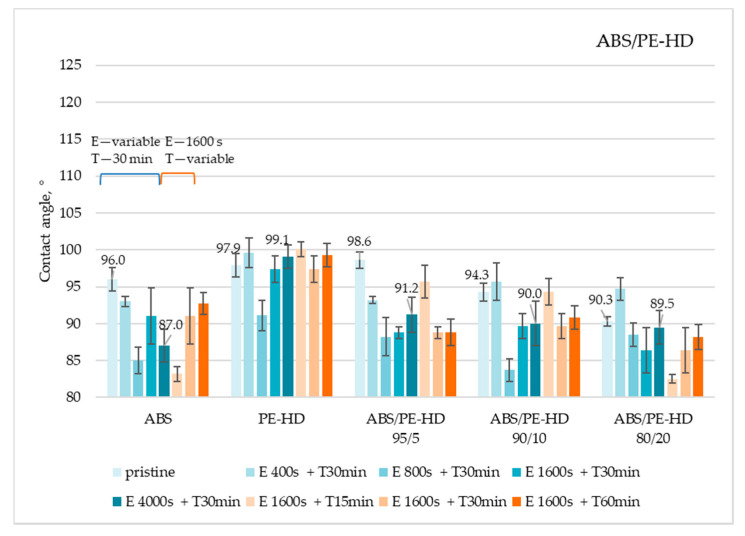
Water contact angles on ABS/PE-HD polymer blends treated under different etching and coating conditions (E—etching; T—CF_x_ coating).

The results of contact angle measurements for ABS/PE-LD and ABS/PE-HD polymer blends are shown in Figure 9 and Figure 10. All data can be found in Supplementary Materials—Table S2.

**Figure 11:**
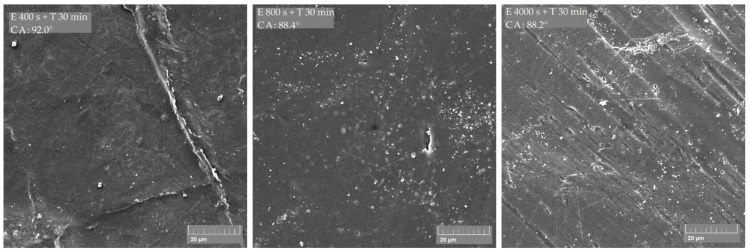
SEM micrographs of untreated ABS/PE-LD 90/10 polymer blend and treated by etching (E) and CF_x_ coating (T); measured contact angle is indicated as CA.

**Figure 12:**
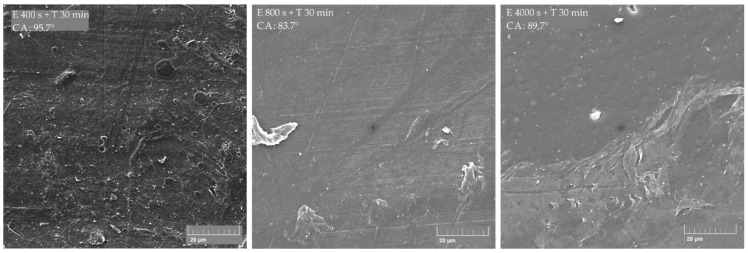
SEM micrographs of untreated ABS/PE-HD 90/10 polymer blend and treated by etching (E) and CF_x_ coating (T); measured contact angle is indicated as CA.

Obtained results showed that, in principle, on all ABS/PE-LD (Figure 9) and ABS/PE-HD (Figure 10) polymer blends no significant increase in the contact angle is achieved by etching and CF_x_ coating. Additionally, an increase in coating time has no impact on gaining a higher contact angle. This is also confirmed with the SEM micrographs of untreated and treated surfaces of ABS/PE-LD and ABS/PE-HD (Figure 11 and Figure 12) where modification of surface roughness is not visible 

**Figure 13:**
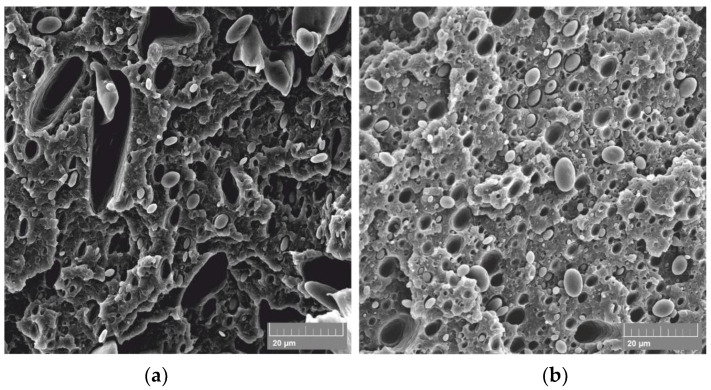
SEM micrographs on the fracture surface of the untreated samples; (**a**) ABS/PE-LD 90/10 and (**b**) ABS/PE-HD 90/10.

In contrast to polymer blends with PLA, it is shown that etching and CF_x_ coating could not significantly change the properties and morphology of the surface in polymer blends with ABS (Figure 11 and Figure 12). No significant change is found on the surface nor the characteristic “island like” structures are created on the surface even though the existence of dispersed domains of PE-LD, as well as PE-HD, in the ABS matrix exists (Figure 13).

Since the treatment of samples with ABS was carried out in the same batch and at the same time as the samples with PLA (which showed a change in the surface morphology of polymer blend) it cannot be concluded that the treatment process was faulty or unsuccessful. Additionally, previous research has shown that surface roughness of both PE and ABS can be modified with argon plasma, i.e., argon ion beam [34,35]. The obtained results for morphology and contact angle of treated ABS/PE-LD and ABS/PE-HD samples may indicate that ABS and PE react equally to plasma, so the etching rate is the same in all areas on the surface, both on the ABS matrix and on the dispersed domain of PE [36], due to which there was no change at the surface structure, and consequently no increase in the contact angle. Additionally, the reason may be in the etching conditions—the strength of the applied plasma and the etching time. Previous studies have shown that the etching time may be insufficient to treat the surface [36] and that the plasma power and etching time exceed the value of critical power and critical processing time followed by a decrease in the total surface roughness [37]. However, to draw a firm conclusion further research is needed. 

**Figure 14:**
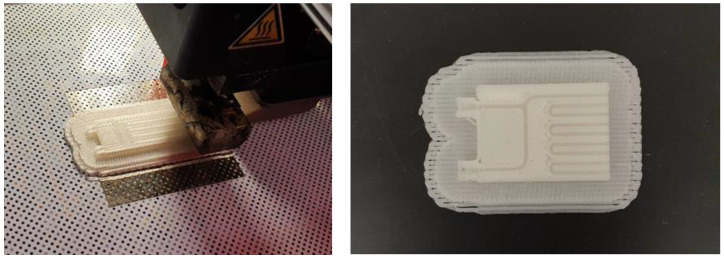
Production process of a microreactor produced by 3D printing and a part of a microreactor with visible microchannels.

In order to prove the possibility of using the PLA/PE-HD 90/10 polymer blend for the manufacturing of microreactors using the 3D printing, a microreactor measuring 48 mm × 14 mm × 3 mm was produced (Figure 14). Since the microreactor is made of an opaque polymer blend it is not possible to see the microchannels inside the reactor when it is finished. In order to see the channels inside the reactor, a part of the microreactor with an open top surface was additionally 3D printed.

## 4. Conclusions

In this research, it has been shown that the modification of the surface morphology of PLA polymer blends can be achieved by ICP etching and fluorocarbon coating. The treated surface of 90/10 PLA/PE-HD showed a contact angle of 121.6°, which is 36° higher than the contact angle measured on the untreated surface. Surfaces that have achieved contact angles higher than 120° have an “island like” surface morphology. The highest contact angle was achieved on a surface with the 4 μm to 8 μm wide islands, where the 6 μm wide islands were the most common. Distance between islands ranged from 10 μm to 20 μm. This surface structure was achieved by etching for 4000 s and CF_x_ coating for 30 min. Samples with higher islands showed higher contact angles, that confirmed that the hydrophobicity also depends on the height of the islands. Furthermore, it was found that etching time significantly impacts the contact angle values and surface morphology of the PLA polymer blends, while the CF_x_ coating time has no significant impact on the surface properties.

Unlike the PLA polymer blends, it has been shown that ICP etching and fluorocarbon coating under the treatment conditions applied in this research cannot significantly change the surface of ABS polymer blends. Additionally, “island like” surface morphology cannot be produced on the ABS polymer blends surfaces under the applied treatment conditions.

## Figures and Tables

**Table 1:** Processing conditions.

Batch Label	Time of Processing	
	Etching (E)	Teflon-Like CF_x_ Coating (T)
E0 + T30	-	30 min
E400 + T30	400 s	30 min
E800 + T30	800 s	30 min
E1600 + T30	1600 s	30 min
E1600 + T15	1600 s	15 min
E1600 + T60	1600 s	60 min
E4000 + T30	4000 s	30 min

## Supplementary Materials

The following are available online at https://www.mdpi.com/1996-1944/13/23/5578/s1, Table S1: Water contact angles on PLA/PE-LD and PLA/PE-HD polymer blends treated under different etching conditions and different coating conditions (E—etching; T—CFx coating), Table S2: Water contact angles on ABS/PE-LD and ABS/PE-HD polymer blends treated under different etching conditions and different coating conditions (E—etching; T—CFx coating).
