# Supplementary material for: Modification of Surface Hydrophobicity of PLA/PE and ABS/PE Polymer Blends by ICP Etching and CFx Coating
Source: Materials (Basel). 2020 Dec 7;13(23):5578. doi: 10.3390/ma13235578 (PMC7729899; doi:10.3390/ma13235578)
Supplement: Supplementary file 1 [file materials-13-05578-s001.pdf]

materials

Supplementary Materials

Modification of Surface Hydrophobicity of PLA/PE and ABS/PE Polymer Blends by ICP Etching and CF_x Coating

Vedrana Lovinčić Milovanović [1,*], Cédric Guyon [2], Ivana Grčić [3], Michael Tatoulian [4], Domagoj Vrsaljko [5,*]

[1] MAXICON Inc., Kružna 22, Zagreb 10000, Croatia
[2] Chimie ParisTech, PSL University, CNRS, Institut de Recherche de Chimie Paris, Paris 75005, France; cedric.guyon@chimieparistech.psl.eu
[3] Faculty of Geotechnical Engineering, University of Zagreb, Varaždin 42000, Croatia; ivana.grcic@gfv.hr
[4] Chimie ParisTech, PSL University, CNRS, Institut de Recherche de Chimie Paris, Paris 75005, France; michael.tatoulian@chimie-paristech.fr
[5] Faculty of Chemical Engineering and Technology, University of Zagreb, Zagreb 10000, Croatia
* Correspondence: vedrana.lovincic@maxicon.hr (V.L.M.); dvrsal@fkit.hr (D.V.)

Received: 21 October 2020; Accepted: 2 December 2020; Published: date

Table S1. Water contact angles on PLA/PE-LD and PLA/PE-HD polymer blends treated under different etching conditions and different coating conditions (E – etching; T – CF_x coating).

		pristine		E 400s + T30min		E 800s + T30min		E 1600s + T15min		E 1600s + T30min		E 1600s + T60min		E 4000s + T30min	
PLA		97,9	± 2,1	101,4	± 1,5	104,0	± 1,4	114,7	± 1,8	105,5	± 4,7	116,9	± 1,8	118,7	± 0,3
PE-LD		95,4	± 1,4	89,4	± 1,0	82,0	± 1,3	88,8	± 2,3	86,2	± 2,1	91,9	± 1,8	89,2	± 2,3
PE-HD		97,9	± 1,6	99,6	± 2,0	91,1	± 2,1	100,1	± 1,0	97,4	± 1,8	99,3	± 1,6	99,1	± 1,6
PLA/PE-LD	95/5	98,9	± 1,6	94,3	± 3,4	103,5	± 2,2	112,8	± 2,7	109,7	± 2,2	114,3	± 1,5	121,0	± 1,4
	90/10	93,7	± 2,2	98,8	± 4,9	102,4	± 1,3	114,1	± 0,8	109,2	± 3,2	-	-	-	-
	80/20	92,5	± 2,8	100,3	± 1,1	99,1	± 3,1	116,6	± 4,3	110,5	± 3,3	115,9	± 2,2	118,9	± 0,8
PLA/PE-HD	95/5	96,6	± 1,4	97,9	± 0,9	100,3	± 1,5	111,8	± 2,0	109,5	± 1,7	115,2	± 1,8	121,0	± 1,6
	90/10	85,6	± 5,3	102,3	± 1,7	103,6	± 3,0	109,4	± 1,9	107,5	± 1,0	111,3	± 2,1	**121,6**	± 0,9
	80/20	90,3	± 1,1	105,8	± 1,9	100,3	± 1,5	103,2	± 2,3	99,3	± 1,4	108,1	± 2,2	113,7	± 1,6

Table S2. Water contact angles on ABS/PE-LD and ABS/PE-HD polymer blends treated under different etching conditions and different coating conditions (E – etching; T – CFx coating).

		pristine		E 400s + T30min		E 800s + T30min		E 1600s + T15min		E 1600s + T30min		E 1600s + T60min		E 4000s + T30min	
ABS		96,0	± 1,6	93,0	± 0,7	85,0	± 1,0	83,2	± 1,0	91,0	± 3,8	92,7	± 1,5	87,0	± 2,2
PE-LD		95,4	± 1,4	89,4	± 1,0	82,0	± 1,3	88,8	± 2,3	86,2	± 2,1	91,9	± 1,8	89,2	± 2,3
PE-HD		97,9	± 1,6	99,6	± 2,0	91,1	± 2,1	100,1	± 1,0	97,4	± 1,8	99,3	± 1,6	99,1	± 1,6
ABS/PE-LD	95/5	95,2	± 0,8	96,0	± 1,3	90,2	± 1,6	92,3	± 1,1	88,8	± 2,0	89,1	± 1,3	86,8	± 3,1
	90/10	87,1	± 2,7	92,0	± 1,1	88,4	± 1,8	84,0	± 2,0	88,2	± 2,4	88,2	± 1,3	88,2	± 1,7
	80/20	-	-	96,6	± 1,2	90,4	± 3,2	82,8	± 2,1	90,4	± 2,8	90,2	± 2,7	88,6	± 1,8
ABS/PE-HD	95/5	98,6	± 1,1	93,2	± 0,5	88,2	± 2,6	95,7	± 2,2	88,8	± 0,8	88,8	± 1,8	91,2	± 2,4
	90/10	94,3	± 1,2	95,7	± 2,5	83,7	± 1,5	94,3	± 1,8	89,7	± 1,7	90,8	± 1,6	90,0	± 3,0
	80/20	90,3	± 0,6	94,7	± 1,5	88,5	± 1,6	82,5	± 0,6	86,4	± 3,1	88,2	± 1,7	89,5	± 2,3
